# A Novel Detection Method of Human Serum Albumin Based on the Poly(Thymine)-Templated Copper Nanoparticles

**DOI:** 10.3390/s17112684

**Published:** 2017-11-21

**Authors:** Mingjian Chen, Xinying Xiang, Kefeng Wu, Hailun He, Hanchun Chen, Changbei Ma

**Affiliations:** School of Life Sciences, Central South University, Changsha 410013, China; chenmingjian@csu.edu.cn (M.C.); bestxxy@126.com (X.X.); kefeng@csu.edu.cn (K.W.); helenhe@csu.edu.cn (H.H.); chenhanchun@csu.edu.cn (H.C.)

**Keywords:** label-free, human serum albumin, copper nanoparticles

## Abstract

In this work, we developed a facile fluorescence method for quantitative detection of human serum albumin (HSA) based on the inhibition of poly(thymine) (poly T)-templated copper nanoparticles (CuNPs) in the presence of HSA. Under normal circumstances, poly T-templated CuNPs can display strong fluorescence with excitation/emission peaks at 340/610 nm. However, in the presence of HSA, it will absorb cupric ion, which will prevent the formation of CuNPs. As a result, the fluorescence intensity will become obviously lower in the presence of HSA. The analyte HSA concentration had a proportional linear relationship with the fluorescence intensity of CuNPs. The detection limit for HSA was 8.2 × 10^−8^ mol·L^−1^. Furthermore, it was also successfully employed to determine HSA in biological samples. Thus, this method has potential applications in point-of-care medical diagnosis and biomedical research.

## 1. Introduction

Quantitative detection of proteins is of great importance in a number of areas, such as chemical and biochemical analyses, immunodiagnostics, and biotechnology [1]. As one of the most abundant blood proteins in plasma, human serum albumin (HSA) plays a very important role in maintaining the oncotic pressure of blood and serves as a carrier for many neutral and weak acidic metabolites and drugs [2,3]. The concentration of HSA in body fluids is almost considered a reliable sign of health [4]. For example, research has shown that there is an intimate connection between cardiovascular disease and kidney damage, and the critical indictor for kidney damage is the concentration of HSA in human urine [5]. Several methods to determine the concentration of HSA have been developed, including visible absorption spectroscopy [6,7,8], electrochemical [9,10], Immunoassays [11,12,13] and High-performance liquid chromatography (HPLC) [14]; however, there are some problems for detecting the concentration of HSA with these methods. For example, Tu and co-workers reported a low-cost method with Immunoassays to detect the HSA [15]. However, this method requires special testing instruments, and a lot of time. Yu Ermolenko et al., reported a methodology for the determination of HSA by electrophoresis, which is sensitive [16]. But the operative cost impedes its application. Therefore, a sensitive, rapid and inexpensive method for the quantitative analysis of HSA is still highly desirable.

As a type of fluorescent probe, DNA-templated copper nanoparticles (CuNPs) have attracted a lot of attention due to some outstanding advantages, such as being cost-effective, environmentally friendly, and highly sensitivity in many areas [17,18,19]. In particular, it can be synthesized more rapidly and at lower cost than AgNCs or AuNPS as a signaling nanomaterial [20]. Thus, CuNPs have been utilized as a label-free, low-cost and facile analysis method in various bio-assays. It has been reported that two kinds of DNA-templated CuNPs, the poly(thymine)-templated CuNPs and the dsDNA-templated CuNPs, have been synthesized [21,22,23]. It is known to us that DNA-templated CuNPs have been used for the detection of many materials, including DNA [24,25], proteins [26,27,28], enzymes [29,30,31], microRNA [32,33], and small molecules [34,35,36].

Here, a fluorescence-based strategy is prepared and applied in detecting the concentration of HSA based on the ploy T-templated CuNPs. HSA will absorb Cu^2+^ because of the strong binding ability between HSA and Cu^2+^, which will prevent Cu^2+^ from being reduced to Cu^0^ by Vitamin c (Vc). As a result, CuNPs can’t be formatted normally. That’s to say, the fluorescence intensity will become lower obviously in the presence of HSA. Thus, the levels of HSA can be determined by monitoring the variation in fluorescence intensity. Furthermore, our method can be used well in detecting HSA in human serum.

## 2. Materials and Methods

### 2.1. Materials and Reagents

HSA was purchased from Sigma Aldrich (St. Louis, MO, USA). Standard solutions of HSA were prepared daily by further dilution of its stock solution (0.03325 g HSA was dissolved in 1 mL ultrapure water and the concentration of HSA is 0.5 mM), which was stored at −20 °C. 3′-(N-morpholino) propanesulfonic acid (MOPS), Vitamin c (Vc) and copper sulfate (CuSO_4_) were purchased from Sinopharm Chemical Reagent (Shanghai, China). The oligonucleotide poly-T, T40, was purchased from Sangon Biological Engineering Technology & Services (Shanghai, China). The DNA sequence is as follows. T40: 5′-TTTTTTTTTTTTTTTTTTTTTTTTTTTTTTTTTTTTTTTT-3′. Ultrapure water (18.2 MΩ·cm) from a Milli-Q water purification system (Millipore Corp, Bedford, MA, USA) was used in all the experiments. The buffer is as follows: MOPS buffer (10 mM MOPS, 150 mM NaCl, and pH 7.5).

### 2.2. Apparatus

All fluorescence measurements were performed on an F-2700 spectrophotometer (Hitachi, Tokyo, Japan) with excitation at 340 nm and emission at 530–650 nm for the poly T-templated CuNPs. The excitation slits and emission slits were set for 10.0 and 10.0 nm, respectively. Each experiment was carried out in a final volume of 100 µL. 

### 2.3. Detection of HSA

For assaying the concentration of HSA, 100 μM Cu^2+^ and different concentrations of HSA were mixed in reaction buffer (10 mM MOPS, 150 mM NaCl, pH 7.5) and incubated for 15 min at room temperature. Then 500 nM T40 and 1 mM Vc were subsequently added to the reaction solution and incubated for 10 min at room temperature before measurement. The total volume of the reaction system is 100 μL.

In order to test the application of the proposed assay in biological systems, spiked samples were prepared by addition of different concentrations of HSA to human serum. 

## 3. Results and Discussion

### 3.1. Verification of the Feasibility of HSA Detection Using Poly T-Templated CuNPs

**Scheme 1:**
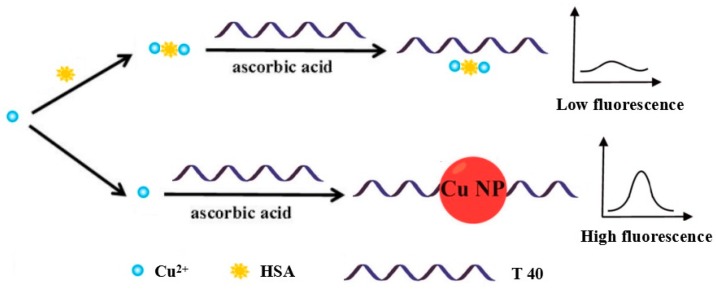
Rapid and sensitive monitoring of HSA based on poly(thymine)-templated copper nanoparticles.

The principle of the detection method is illustrated in Scheme 1. Our experimental method selected Poly T strand (T40) as the template for CuNPs formation [37]. Under normal circumstances, Cu^2+^ could be reduced to Cu^0^ by Vc, which reacts with T40 to form the poly T-templated CuNPs and generates a strongly fluorescent signal. However, in the presence of HSA, it will absorb the Cu^2+^ to prevent the reduction reaction by ascorbic acid. As the result, poly T-templated CuNPs could not be formed after adding T40 to the reaction solution, which meant that the mixture could not generate nearly the same fluorescence intensity. Therefore, we were able to detect the concentration of HSA by monitoring the variation in fluorescence intensity.

**Figure 1:**
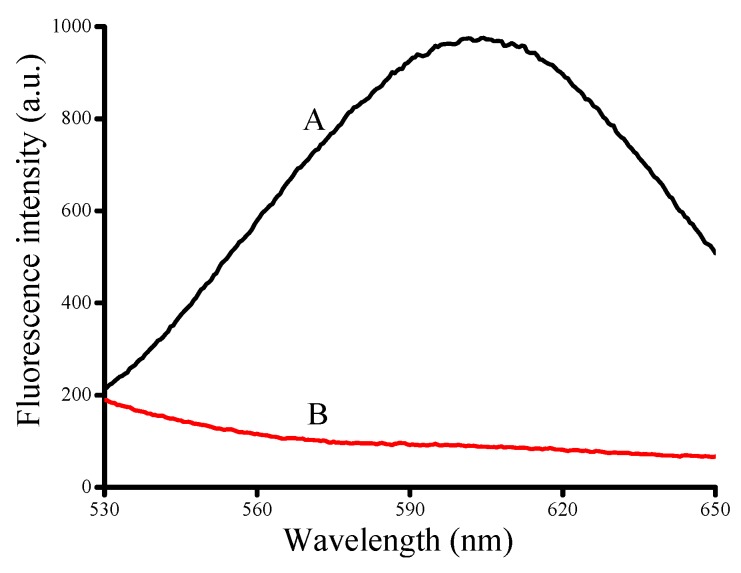
The feasibility of the proposed method. (**A**) Synthesis of CuNPs with 500 nM T40, 100 μM CuSO_4_ and 1 mM Vc; (**B**) 500 nM T40, 100 μM CuSO_4_ and 1 mM Vc were incubated for 10 min at room temperature after prep-incubation of the CuSO_4_ with 5 μM HSA for 15 min at room temperature.

The feasibility of the proposed method was verified. It could apparently observe strong levels of fluorescence at 340 nm excitation when T40, Cu^2+^ and Vc were added to the buffer (Figure 1A). In contrast, we could see that it didn’t form any fluorescence after adding HSA (Figure 1B). In summary, the results of this experiment demonstrated the feasibility of the proposed strategy for detection of HSA.

### 3.2. Optimization of Experimental Conditions

**Figure 2:**
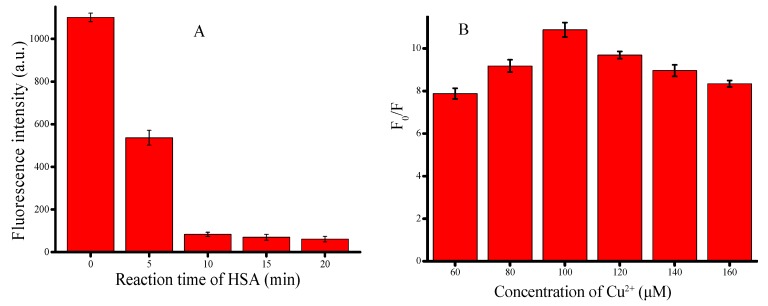
Optimization of experimental conditions. (**A**) Changes in fluorescence intensity with reaction time of HSA (0, 5, 10, 15, 20 min); (**B**) Changes in fluorescence intensity with different concentrations of CuSO_4_ (60, 80, 100, 120, 140, 160 μM). Error bars were estimated from three replicate measurements.

After verifying the feasibility of the proposed method, we investigated the effects of different concentrations of Cu^2+^ and the reaction time of HSA with Cu^2+^ on the sensitivity and fluorescence response characteristics of the proposed method. Firstly, we conducted an experiment about the reaction time of HSA with the Cu^2+^. After a series of fluorescence measurements, it could be found that the fluorescence intensity reduced with the reaction time and reached a steady value after 15 min (Figure 2A). In order to get more stable experimental results, 15 min was considered as the optimal reaction time between HSA and Cu^2+^.

Then, the effect of different concentrations of Cu^2+^ was investigated. As can be seen in Figure 2B, the optimum rate of increase in fluorescence intensity (F_0_/F, where F_0_ represented the fluorescence intensity without HSA and F for the fluorescence intensity with HSA) occurred when the concentration of Cu^2+^ was 100 μM. Thus, 100 μM Cu^2+^ was used for subsequent experiments. 

### 3.3. Quantitative Measurement of HSA

**Figure 3:**
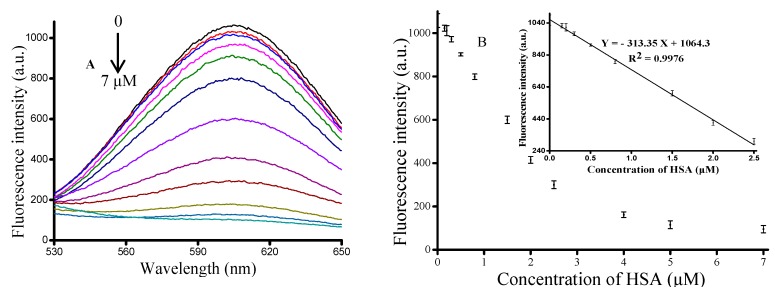
(**A**) Fluorescence emission spectra of the concentration of HSA (0, 0.15, 0.2, 0.3, 0.5, 0.8, 1.5, 2, 2.5, 4, 7 μM). Optimized assay conditions were used; (**B**) Graph depicting the changes in fluorescence output at 610 nm as a function of HSA concentration. Inset: Linear relationship between fluorescence intensity and low HSA concentrations. Error bars were estimated from three replicate measurements.

In order to evaluate the sensitivity of the proposed method, we chose a series concentration of HSA ranging from 0 to 7 μM (0, 0.15, 0.2, 0.3, 0.5, 0.8, 1.5, 2, 2.5, 4, 5, 7 μM). As seen in Figure 3A, we found that the fluorescence intensity at 610 nm dynamically decreased with an increase in the concentration of HSA. Figure 3A shows the relationship between fluorescence intensity and the concentration of HSA. The inset of Figure 3B showed that the fluorescence intensity had a linear relationship (R^2^ = 0.9976) with HSA concentration in the concentration range of 0.15–2.5 μM, and the regression equation was Y = −313.35 X + 1064.3, where Y was the fluorescence intensity at 610 nm and X was the HA concentration. The limit of detection (LOD) of the proposed strategy was estimated to be 0.082 μM, which was lower and more rapid than the other methods [38,39,40]. Therefore, a simple, rapid, effective and sensitive method of HA determination was established. 

### 3.4. Study of Interferences

**Figure 4:**
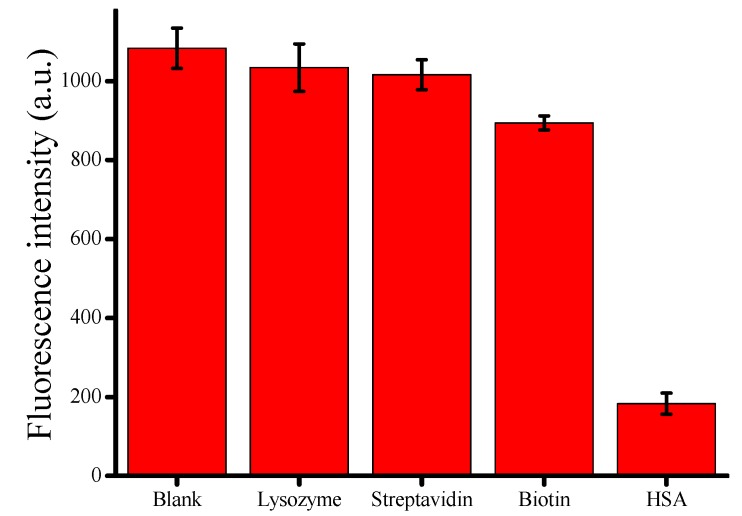
Fluorescence intensity of CuNPs in the presence of lysozyme, streptavidin, biotin and HSA under optimized concentrations. Error bars were estimated from three replicate measurements.

We tested several proteins, such as lysozyme, streptavidin and biotin, by the proposed assay under the optimized concentrations to investigate the selectivity. As seen in Figure 4, we found that none of the proteins influenced the synthesis of CuNPs except HSA. That’s to say, the proposed assay was specific for the detection of HSA, showing a great potential in biological samples.

**Figure 5:**
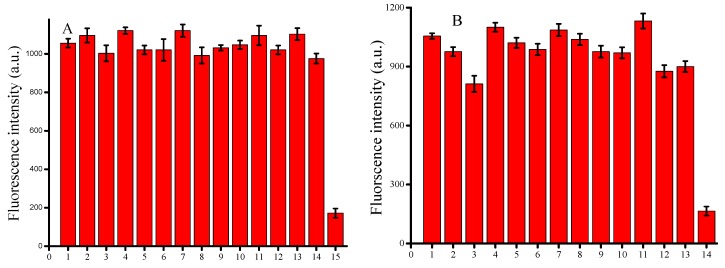
(**A**) Fluorescence emission spectra of the reaction system with HSA (7 μM) and various ions (7 μM) under the optimized concentrations. 1, Blank; 2, K^+^; 3, Mn^2+^; 4, Ca^2+^; 5, Na^+^; 6, Mg^2+^; 7, Li^+^; 8, Al^3+^; 9, Zn^2+^; 10, Hg^2+^; 11, Cl^−^; 12, NO_3_^−^; 13, SO_4_^2−^; 14, CO_3_^2−^; 15, HAS; (**B**) Fluorescence emission spectra of the reaction system with HSA (7 μM) and various organics (7 μM) under the optimized concentrations. 1, Blank; 2, arginine; 3, glutamic; 4, glycine; 5, isoleucine; 6, leucine; 7, lyscine; 8, valine; 9, proline; 10, alanine; 11, glucose; 12, sucrose; 13, carbamide; 14, HSA.

Organic and inorganic compounds typically occurring in human serum were studied as potential interferents. It was considered not to interfere when various kinds of ions and amino acids caused minimal changes in the fluorescence intensity. The results are shown in Figure 5A,B. Therefore, our method has a wider linear range for detecting the HSA in human serum.

### 3.5. Application of the Proposed Assay in Biological Systems

In order to evaluate the practical application of the proposed sensing platform, we attempted to detect HSA in 1% human serum. We added different concentrations of HSA into the diluted blood samples and detected it again with the proposed method. The results are shown in Table 1: the recovery rates of different concentrations of HSA in human serum were 97.0% for 0.86 μM, 101.0% for 1.36 μM, and 99.7% for 1.86 μM. These results indicated that the method might have practical applications for HSA detection in biological systems.

## 4. Conclusions

In summary, we have successfully demonstrated a fluorescence method for HSA detection based on the poly T-templated copper nanoparticles. The proposed method exhibits high sensitivity to HSA, with a detection limit of 8.2 × 10^−8^ mol·L^−1^ under the optimized conditions. Besides, this method is simple and cost-effective, without any labels or complicated operations. The proposed strategy was also successfully applied in detection of HSA in serum samples, and satisfactory results were obtained. We envision that our strategy based on poly T-templated copper nanoparticles for detection of HSA may be applied in in point-of-care medical diagnosis.

## Figures and Tables

**Table 1:** Recovery of HSA in diluted human serum using the proposed method.

Sample	HSA in Plasma (μM)	Added (μM)	Detected (μM)	Recovery (%)
1	0.36	0.5	0.834 ± 0.036	97.0
2	0.36	1	1.374 ± 0.064	101.0
3	0.36	1.5	1.854 ± 0.094	99.7
